# Clinical Acceptability of a Quality Improvement Program for Reducing Cardiovascular Disease Risk in People With Chronic Kidney Disease in Australian General Practice: Qualitative Study

**DOI:** 10.2196/55667

**Published:** 2024-11-13

**Authors:** Caroline McBride, Barbara Hunter, Natalie Lumsden, Kaleswari Somasundaram, Rita McMorrow, Douglas Boyle, Jon Emery, Craig Nelson, Jo-Anne Manski-Nankervis

**Affiliations:** 1 Department of General Practice and Primary Care, The University of Melbourne, Melbourne, Australia; 2 Western Health Chronic Disease Alliance, Western Health, Sunshine, Australia; 3 Department of Nephrology, Western Health, Sunshine, Australia; 4 Department of Medicine, The University of Melbourne, Melbourne, Australia

**Keywords:** clinical decision support, general practice, GP, primary care, family medicine, general medicine, family physician, implementation science, chronic kidney disease, CKD, nephrology, nephrologist, chronic disease, cardiovascular risk, cardiology, quality improvement, EHR, electronic health record, clinical software

## Abstract

**Background:**

Future Health Today (FHT) is a technology program that integrates with general practice clinical software to provide point of care (PoC) clinical decision support and a quality improvement dashboard. This qualitative study looks at the use of FHT in the context of cardiovascular disease risk in chronic kidney disease (CKD).

**Objective:**

This study aims to explore factors influencing clinical implementation of the FHT module focusing on cardiovascular risk in CKD, from the perspectives of participating general practitioner staff.

**Methods:**

Practices in Victoria were recruited to participate in a pragmatic cluster randomized controlled trial using FHT, of which 19 practices were randomly assigned to use FHT’s cardiovascular risk in CKD program. A total of 13 semistructured interviews were undertaken with a nominated general practitioner (n=7) or practice nurse (n=6) from 10 participating practices. Interview questions focused on the clinical usefulness of the tool and its place in clinical workflows. Qualitative data were coded by 2 researchers and analyzed using framework analysis and Clinical Performance Feedback Intervention Theory.

**Results:**

All 13 interviewees had used the FHT PoC tool, and feedback was largely positive. Overall, clinicians described engaging with the tool as a “prompt” or “reminder” system. Themes reflected that the tool’s goals and clinical content were aligned with clinician’s existing priorities and knowledge, and the tool’s design facilitated easy integration into existing workflows. The main barrier to implementation identified by 2 clinicians was notification fatigue. A total of 7 interviewees had used the FHT dashboard tool. The main barriers to use were its limited integration into clinical workflows, such that some participants did not know of its existence; clinicians’ competing clinical priorities; and limited time to learn and use the tool.

**Conclusions:**

This study identified many facilitators for the successful use of the FHT PoC program, in the context of cardiovascular risk in CKD, and barriers to the use of the dashboard program. This work will be used to inform the wider implementation of FHT, as well as the development of future modules of FHT for other risk or disease states.

## Introduction

### Background

The 2011‐12 Australian Health Survey revealed that 10% of Australian adults had biochemical signs of chronic kidney disease (CKD) [1]. CKD is a significant risk factor for cardiovascular disease [2], which is Australia’s leading cause of death [3]. Early intervention can slow the deterioration in kidney function and reduce the risk of cardiovascular complications [4]. Australian CKD guidelines recommend the use of angiotensin-converting enzyme inhibitors (ACEis) or angiotensin-receptor blockers (ARBs) in the presence of albuminuria or hypertension (defined as a blood pressure above 130/80 mm Hg), and statins are recommended for everyone with CKD over the age of 50 years or for younger individuals with CKD in the presence of comorbidities [5].

CKD is underrecognized and undertreated in Australian general practice. One study found that 76.8% of people with biochemical results consistent with CKD did not have the diagnosis recorded in their medical file [6], and another found that only 65% of people with a formal diagnosis of CKD were prescribed an ACEi/ARB while 56% were prescribed a statin [7]. A known barrier to achieving quality improvement in chronic disease is the lack of timely and straightforward access to clinical guidelines [8,9]. Electronic clinical decision support tools aim to address this barrier by integrating with clinical records to automatically provide key information from appropriate clinical guidelines.

### Prior Work

The Future Health Today (FHT) program is a general practice quality improvement technology platform developed by the University of Melbourne and Western Health. FHT integrates with the electronic medical record to provide 2 components: a clinical decision support tool active at the point of care (PoC) and a web-based dashboard that facilitates practice-wide audit. Screenshots of FHT are available in Multimedia Appendix 1. FHT was developed using a service design approach, whereby clinicians were involved throughout an iterative technical development process [10]. It underwent optimization at 12 general practice clinics, using guidelines for CKD, cardiovascular disease, and cancer prevention. A qualitative optimization study for the cancer recommendations has been described elsewhere [11]. A cluster randomized controlled trial was completed in 2022 (trial registration: ACTRN12620000993998) with the aim of exploring whether the FHT platform increased the prescription of ACEis/ARBs and statins in people with CKD at high cardiovascular disease risk [12]. This paper reports on barriers and facilitators of clinical performance improvement as reported by clinicians themselves.

## Methods

### Study Setting

General practice provides primary care to more than 8 in 10 Australians each year [13] and operates on a fee-for-service model, with rebates available through Medicare, the health care insurance scheme funded by the Australian Government Department of Health and Aged Care. General practices may bulk bill (payment covered in full by Medicare), privately bill (patients pay a fee in excess of the Medicare rebate), or mixed bill (variably bulk bill or privately bill patients).

The FHT trial was conducted in 39 general practices in Victoria and 1 general practice in Tasmania, Australia. In summary, clinics were randomized into 2 arms: cardiovascular risk reduction in people with CKD or identification of cancer risk, with each group acting as the control for the other [12]. Twenty-one general practices were allocated to the CKD arm, with 19 practices completing the trial. The trial ran from October 2021 to September 2022. The CKD algorithm was deactivated in December 2021 after errors in algorithm deployment were noted and was reactivated in February 2022. The interviews analyzed in this paper occurred between May and July 2022. The state of Victoria was subject to a COVID-19 pandemic declaration throughout the duration of the trial.

### Study Design

This qualitative implementation study focuses on the clinical experience and use of FHT for medication management to reduce cardiovascular risk in people with CKD. Semistructured interviews were conducted with general practitioners (GPs) and practice nurses (PNs) participating in the CKD intervention arm of the FHT trial.

### Participants

Participating clinics were recruited via VicREN (Victorian Primary Care Practice-Based Research and Education Network) at the Department of General Practice and Primary Care, The University of Melbourne, and The University of Tasmania’s Northern Tasmania General Practice-based research network. Each practice was asked to nominate a practice champion at the beginning of the trial; this tended to be a PN or practice manager. The practice champion was requested to assist with the recruitment of GPs and PNs to participate in one-on-one interviews held between 8 and 10 months into the trial. Recruitment was also facilitated by the FHT trial coordinator. Potential participants were emailed a plain-language statement and consent form to complete if they were interested in participating. Participants were reimbursed with a AUD $50 (US $33.69) debit card voucher.

### Data Collection

Interviews occurred over the phone and were recorded for transcription. There were 3 interviewers: CM, NL, and KS. CM is an academic GP registrar, NL is a research fellow, and KS is a research assistant. All are female and are affiliated with the University of Melbourne. NL acted in a liaison role for practices during the trial. An interview guide (Multimedia Appendix 2) was developed to focus on questions regarding the clinical usefulness of the recommendations, impact on clinical workflows, and perceived change in clinical performance.

### Data Analysis

Interview data were analyzed using Clinical Performance Feedback Intervention Theory (CP-FIT), a theory for designing, implementing, and evaluating feedback specifically in the health care context [14]. The theory proposes 42 variables that influence a feedback cycle via 7 mechanisms. Each step in the cycle is vital for successful feedback to occur. Each interview was independently coded by CM and BH (a qualitative researcher) using a combination of NVivo (Lumivero) and Microsoft Excel. Any discrepancies in coding were discussed and a consensus was reached.

### Ethical Considerations

The overall FHT trial protocol was approved by the Department of General Practice University of Melbourne Human Ethics Advisory Group and the Faculty of Medicine, Dentistry, and Health Sciences Human Ethics Sub-Committee. The ethics ID is 2056564. These ethics committees use the National Statement on Ethical Conduct in Human Research published by the Australian Government National Health and Medical Research Council.

## Results

### Overview

A total of 13 interviews were held, with staff from 10 clinics. The demographic and clinic details of the 7 GP and 6 PN participants are summarized in Table 1. Participants were assigned a code based on their role (GP or PN) and their clinic’s allocated number within the trial. All 10 clinics were located in the state of Victoria and varied in their location (regional Victoria or metropolitan Melbourne) and billing policy (mixed or bulk billing). The sample size aimed to gather perspectives from as many staff at the 19 intervention practices as possible but was limited by the response rate. A total of 5 practices did not respond to the request for interview, 1 canceled citing personal reasons, and 1 canceled citing lack of use of FHT. Duration of interviews ranged from 14 to 39 minutes (average 21 min).

All interviewees had used the FHT PoC tool (N=13), with only 7 interviewees having experience using the FHT dashboard. The 5 GPs not using the dashboard were not aware of it, were not able to access it due to technological issues, or had not had time to use it. Five of the PNs had used the dashboard, but none had used it to recall patients.

**Table 1.** Interview participant characteristics.

Participant	Sex	Location	Billing policy	Use of FHT^a^
GP7A^b^	Male	Regional	Mixed	PoC^c^
GP7B	Male	Regional	Mixed	PoC
GP9	Male	Metro	Bulk billing	PoC and dashboard
GP22	Female	Metro	Mixed	PoC
GP23	Male	Regional	Mixed	PoC
GP31	Female	Metro	Bulk billing	PoC and dashboard
GP38	Male	Metro	Mixed	PoC
PN5^d^	Female	Metro	Mixed	PoC and dashboard
PN6	Female	Metro	Mixed	PoC and dashboard
PN18	Male	Metro	Bulk billing	PoC
PN19	Female	Metro	Bulk billing	PoC and dashboard
PN22	Female	Metro	Mixed	PoC and dashboard
PN23	Female	Regional	Mixed	PoC and dashboard

a FHT: Future Health Today.

b GP: general practitioner.

c PoC: point of care.

d PN: practice nurse.

### Interview Findings: The CP-FIT Feedback Cycle

**Figure 1.**
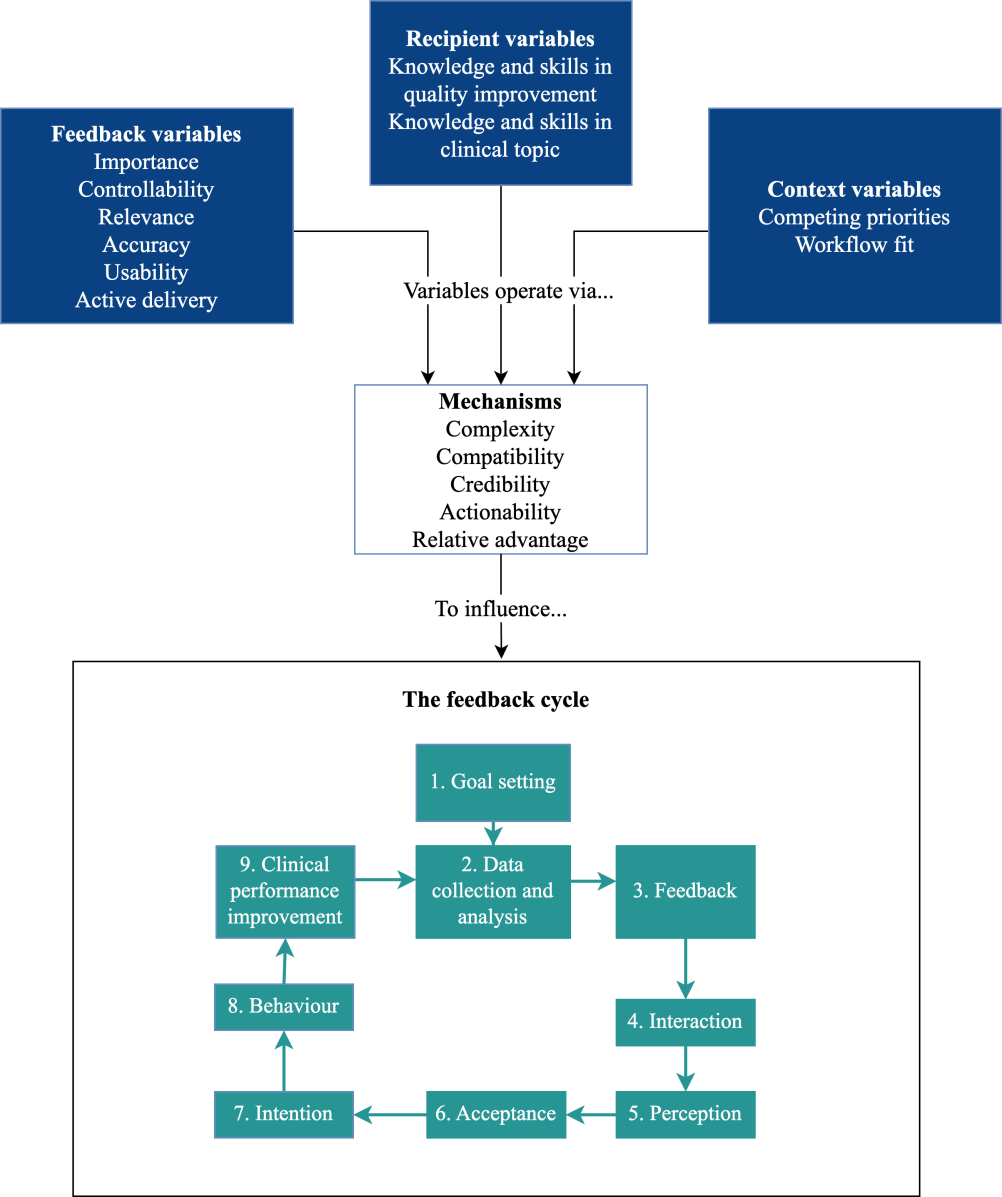
Clinical Performance Feedback Intervention Theory variables, explanatory mechanisms, and feedback cycle (adapted from Brown et al [14], which is published under a Creative Commons Attribution 4.0 International License [15]).

Results were mapped to the CP-FIT feedback cycle. An adapted version of CP-FIT is shown in Figure 1, with variables and mechanisms relevant to this study included in the diagram. In total, 10 explanatory variables and 5 mechanisms were identified as influencing how clinicians used FHT.

### Interview Findings: The PoC Tool

**Figure 2.**
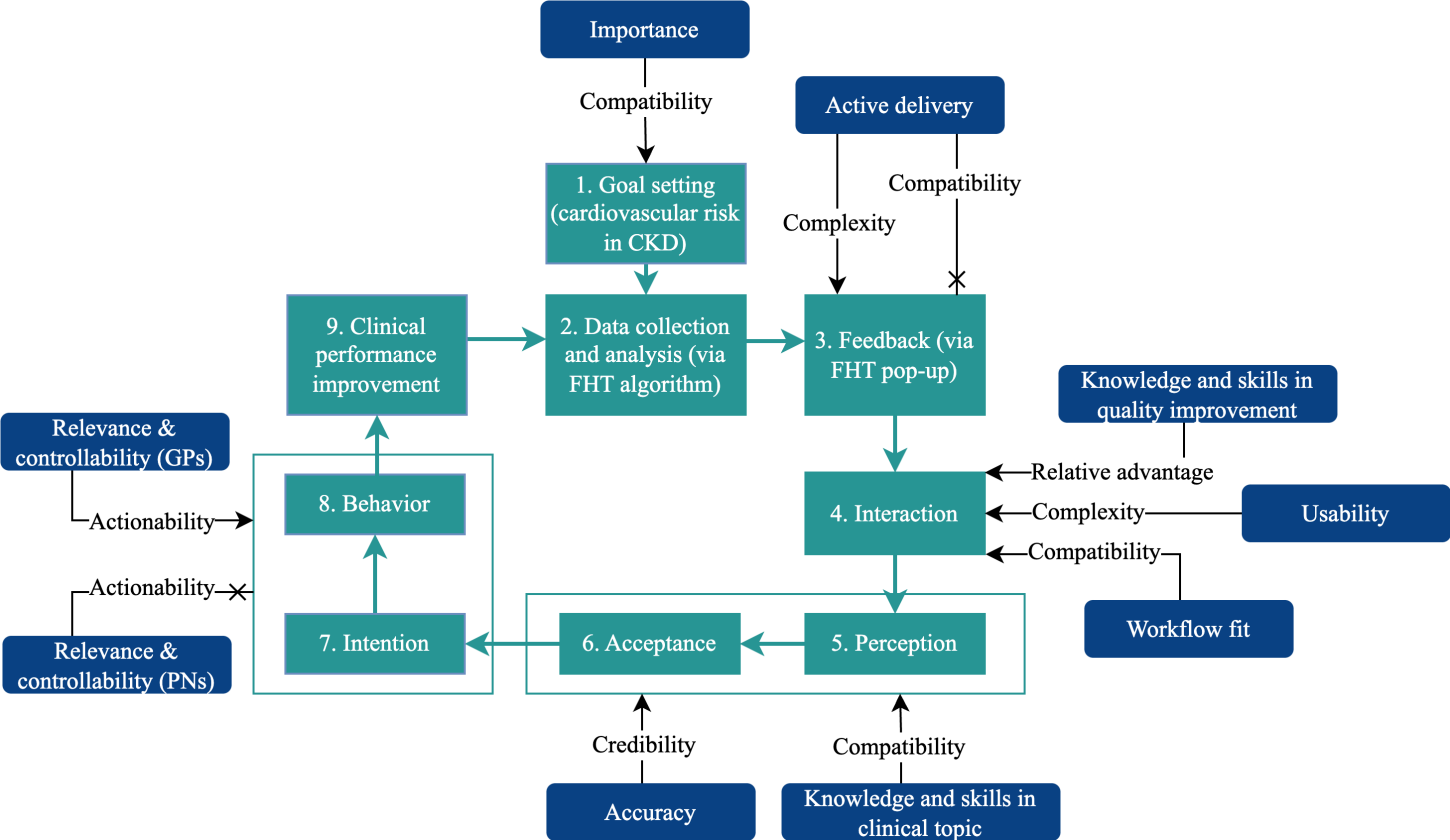
The Clinical Performance Feedback Intervention Theory feedback cycle when using the point of care tool (adapted from Brown et al [14], which is published under a Creative Commons Attribution 4.0 International License [15]). CKD: chronic kidney disease; FHT: Future Health Today; GP: general practitioner; PN: practice nurse.

Results for the PoC tool are presented in sequential order following the CP-FIT feedback cycle as shown in Figure 2, with key quotes to illustrate themes. Some steps of the feedback cycle are presented concurrently, reflecting that participants described these processes occurring simultaneously.

#### Goal Setting: Importance

It was universally agreed that CKD is an area of importance in general practice. Clinicians reflected on how CKD is underrecognized and undertreated.

It’s certainly an area that seems to be pretty poorly documented in the patient’s records and then consequently not always particularly well treated either.

[GP7A]

It’s actually really important, because it’s – probably 10 per cent of the clinic patients have chronic kidney disease, according to the statistics and probably I don’t have all of them already coded in my computer

[GP31]

#### Data Collection and Analysis

Step 2 occurred automatically via the FHT software program, without needing intervention by clinicians.

#### Feedback: Active Delivery

Active delivery of feedback via the PoC pop-up facilitated ease of use. Put simply:

It’s just there. It just pops up. I read it.

[PN5]

The method of delivery was designed to not be intrusive, and this is consistent with how it was perceived.

It’s quite a minimal popup. Well, it has to be big enough that you see it, but it’s small enough that it doesn't just take over the whole of the screen.

[GP7A]

Two participants identified concerns about notification fatigue.

[Interviewer: have people been receptive to learning how to use it?] Yeah. But also…that’s one more thing we have to deal with. There has been a little bit of reluctance.

[PN5]

Any decision support software…you get notification fatigue

[GP7B]

#### Interaction: Knowledge and Skills in Quality Improvement, Usability, and Workflow Fit

GPs demonstrated a keen interest in quality improvement and proactively reviewed information from the PoC tool to facilitate this.

It’s always good to review your management.

[GP31]

There will be a patient that they are not on what’s recommended just because of an oversight there. So I think that it’s a good double check.

[GP23]

Clinicians found the PoC tool to be usable because of its information clarity and lack of complexity.

They are useful because they’re clear, or clear-cut and they’re not a big swath of information coming through, and that gives you an opportunity to go into the resources as you need to.

[GP7A]

For GPs, the PoC tool was compatible with existing workflows. Most GPs reported reading and verifying the information in the PoC tool prior to bringing the patient into their consulting room, as part of their usual preconsultation file review. This process was described as very quick (GP23, GP31), and the recommendations were able to be actioned within their standard consultation structure.

I usually familiarise myself with the patients before calling them, so I go through the history, recalls, results or whatever, including if there was any popups

[GP31]

It’s pretty straightforward. The discussion goes something like, “so Mrs. Jones, just looking at your recent renal function, the current recommendations is that we add a statin or you should be on an ACE or ARB. what do you think about that?” They usually say, well, what do you think? And we go down that path of shared decision making.

[GP7B]

PNs described more variation in how they interacted with the PoC tool and integrated it into their workflows. Two described using the program opportunistically (PN5, PN19) during consultations or when reviewing existing recalls. One reviewed the recommendations only when the patient was booked for a long appointment such as a care plan or health assessment. Practices nurses mostly acted on the recommendations by documenting in the patient file or directly discussing with the patient’s GP.

It’s for patients who have long appointments. For example, they’re in for a care plan or a health assessment wherein we actually review and check the file for a longer period of time. Unlike those who are only here for, let’s say, vaccinations where they will be in and out of the treatment room and we don’t really have that much time to review and study.

[PN22]

I usually will just alert the doctor and then…the doctor will, based on their own view and they would decide whether or not to follow the advice

[PN6]

#### Perception and Acceptance: Knowledge and Skills in Clinical Topic and Accuracy of Recommendations

When asked directly, each GP reported a good pre-existing knowledge of CKD; none of the information in the PoC tool was new or surprising. They expressed confidence in interpreting and actioning the recommendations in the context of each patient.

Guidelines are guidelines. That’s exactly what they are. They’re not - they don’t actually always - are not always appropriate for the person sitting in front of you. we go down that path of shared decision making.

[GP7B]

Every patient that I have I have a look at the recommendations to see what their recommendations are and if it comes up in orange or that I consider their recommendations and follow them unless there’s a reason not to. I review my medication and have a good think about it.

[GP23]

Most PNs reported less confidence with CKD management in general but were empowered by the PoC tool to learn more.

I don’t think I know that area so well, yeah, so it is quite useful to have this pop-up icon.

[PN6]

GPs largely found the recommendations to be accurate. The process for verifying information was straightforward and they felt comfortable discounting recommendations when appropriate. The format of clinical data storage and the way data were recorded in the electronic medical record were identified as potential sources of inaccuracy.

I think it seems to be pretty accurate most of the time…normally I will go having a look through the most recent result eGFR and any albumins that are there and just see if there is anything that does meet the criteria for a diagnosis. Then if not, then I’ll leave it.

[GP7A]

I think a lot of them are on it. It just wasn’t documented in the right place…one of the limitations in this clinic in particular is that [GP] handwrites scripts

[PN19]

#### Intention and Behavior: Usability, Relevance, and Controllability

Themes related to controllability and relevance diverged according to role, due to the recommendations having a focus on medication and prescribing. GPs found that the PoC recommendations were well within their scope and control, and most GPs found them relevant to their patient population.

Every time, if it came up with a recommendation, I would act on it to decide whether they should be on it or whether there’s a reason not to.

[GP23]

In contrast, PNs described how the PoC had limited direct relevance to their role, which excludes prescribing.

I can’t prescribe anything, and a lot of it is about prescribing

[PN5]

#### Clinical Performance Improvement

Overall, these themes reflected the view that the PoC tool was seen as a “prompt” or “reminder” that reinforced pre-existing knowledge in a straightforward manner consistent with usual practice.

It’s a nice little way of reminding people to do it really…. I think just having it coming up is always reminding me to check what are the best practice principles, things like the ACE and ARB.

[GP7A]

I think it’s just a good prompt because it does pop up there, when you can get really busy.

[PN19]

One GP reflected on how the presence of FHT had the effect of encouraging more general clinical performance improvement.

It certainly does focus particularly in general consults on preventative health and flags that as an issue to discuss and might then prompt me to not only look at the CKD, but make sure they’ve had a recent blood pressure and height and weight and make sure their cancer screening is up to date; all of those sorts of things as well.

[GP7A]

### Interview Findings: The Dashboard Tool

**Figure 3.**
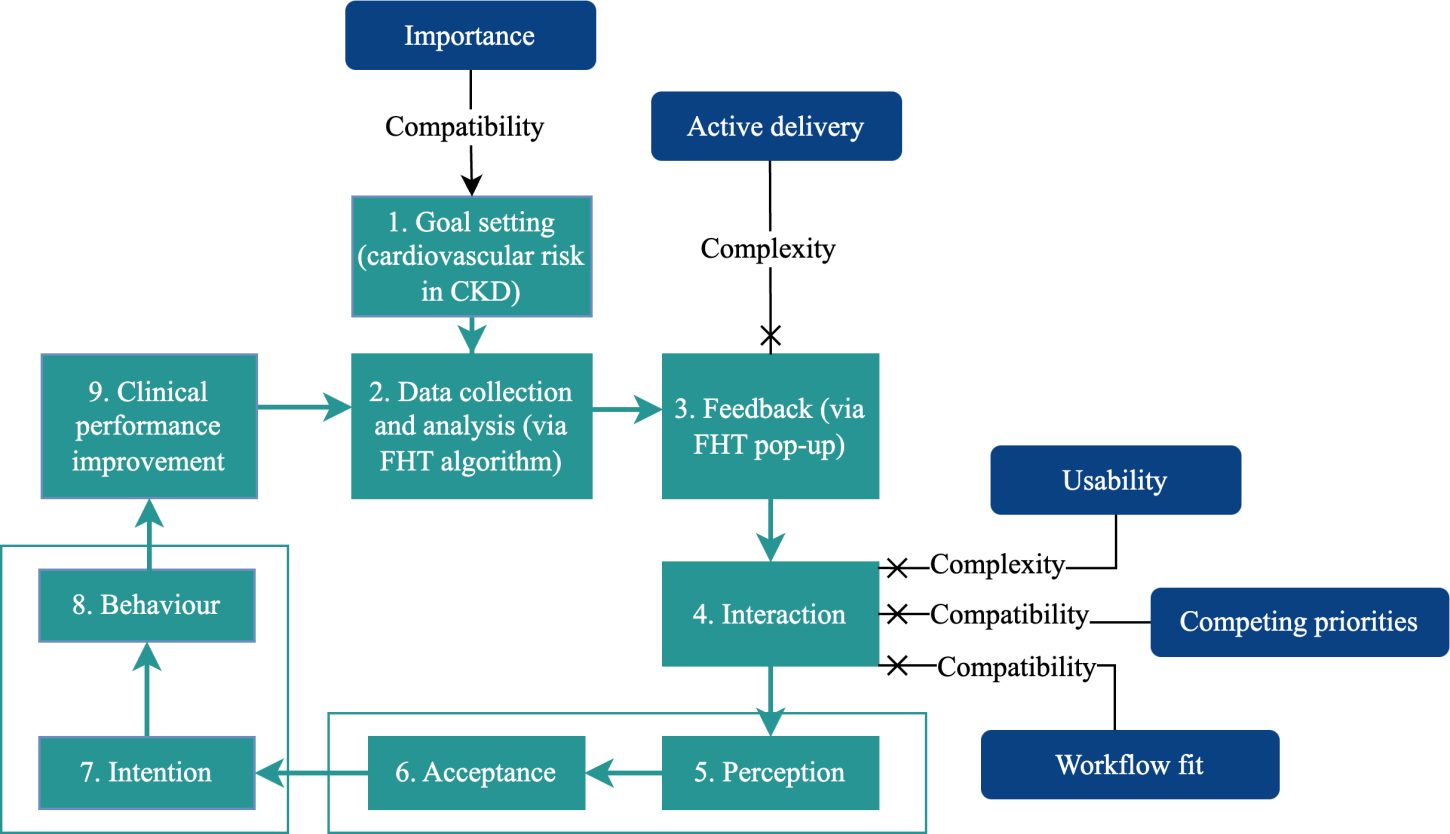
The Clinical Performance Feedback Intervention Theory feedback cycle when using the dashboard tool (adapted from Brown et al [14], which is published under a Creative Commons Attribution 4.0 International License [15]). CKD: chronic kidney disease; FHT: Future Health Today.

Results for the dashboard tool are shown in Figure 3. Feedback cycle steps 1 and 2 (goal setting and data collection) operated in the same manner as the PoC tool. However, participants became “stuck” at either steps 3 or 4.

#### Feedback: Active Delivery

Use of the dashboard was significantly limited by its lack of active delivery; multiple clinicians were not aware it existed.

#### Interaction: Usability, Competing Priorities, and Workflow Fit

Clinicians who had used the dashboard reported some difficulty learning how to navigate the tool, and use of the dashboard was largely incompatible with existing workflows. This was exacerbated by time constraints associated with additional clinical tasks and workforce pressures, particularly with the impacts of the COVID-19 pandemic.

I get a little bit lost still in [the dashboard].

[PN19]

A lot of it is just timing. We’re still so busy. I would love to have a bit more time to sit down and just work through things. We’re starting to do a little bit now but, really, it’s just time.

[PN5]

With the Pfizer vaccines and all that we are inundated with work…COVID has really taken over

[GP22]

## Discussion

### Principal Results: The PoC Tool

In this qualitative study exploring the implementation of FHT using CP-FIT, we found that the FHT PoC tool was well received by clinicians and facilitated guideline-informed care. When mapped to the CP-FIT feedback cycle in Figure 2, we could see that they were able to move rapidly through all steps of the process, facilitated by the variables identified. GPs found that the tool was compatible with their quality improvement goals and clinical workflows; was simple to use; and provided credible, clinically useful information. This was supported by their pre-existing confidence in managing CKD, so the tool was seen as a quick reminder system that prompted further personalized review and discussion. Clinicians in our study worked across a range of location and billing styles, but all described similar workflows and clinical knowledge that supported uptake of the tool. This concept of a clinical decision support tool working most effectively as an “aid-memoire” is supported by existing literature [16]. PNs had a slightly different experience given their self-reported lesser knowledge of CKD and limited role in medication prescribing, but nonetheless, they described the PoC tool as useful and simple to use.

### Principal Results: Dashboard Tool

Use of the FHT dashboard tool was limited. Most GPs did not get to the “feedback” stage of accessing the dashboard, and most PNs did not progress past initial interactions with the software. During the co-design phase, clinicians had expressed a desire for FHT to facilitate both planned and spontaneous interactions [10], but in practice, most interactions occurred spontaneously. The functions available in the dashboard did align with the goals described by clinicians in both the co-design phase and this implementation study, but this was insufficient to overcome competing clinical priorities, particularly the lack of time and staff resources in the context of the COVID-19 pandemic.

The barriers to the use of the dashboard tool stood in clear contrast to the facilitators for the PoC tool. Whereas the PoC tool was simple to access by virtue of being a “pop-up,” the dashboard required clicking away from the patient file. The visual design of the PoC tool and wording of the recommendations were designed with simplicity and conciseness as a priority. By contrast, the dashboard tool has multiple aspects of functionality, allowing for extensive customization (Multimedia Appendix 1). The few clinicians who reached the tool described being overwhelmed or not having enough time to learn how to use the tool.

The nature of Australian general practice funding tends to incentivize opportunistic rather than systematic preventative health activities [17]. Medicare uses a fee-for-service model, whereby patients receive a rebate for attendance either in person or via telehealth/telephone. Alternative payment mechanisms have been proposed that allow for more of a focus on proactive, coordinated care that includes funding non–face-to-face care, such as that required to interact with tools like the FHT dashboard in any meaningful way [17].

### Limitations

All clinicians we interviewed had used FHT in their workplace; by nature of agreeing to be interviewed, they were able to describe the enablers that led them to progress along the CP-FIT feedback pathway. Respondent bias is a limitation of our study, as those who feel most strongly are most likely to speak with us. We also acknowledge that the sample size may not encompass the experiences within the diverse general practice context. However, given the impact of the COVID-19 pandemic on primary care, we are grateful to the 13 participants who took the time to speak with us. The barriers remain unknown for clinicians who did not use FHT, although they can be hypothesized, as discussed below.

While this study focused on the implementation of FHT from the clinical perspective, there are of course other important considerations. In particular, other studies have identified concerns related to the financial costs of staff time and the software itself [18].

### Comparison With Prior Work

Many electronic quality improvement programs in current use in Australian general practice have not been evaluated in a research context, and as such, there is a lack of data exploring implementation factors for these programs. The Australian Government Department of Health has recognized the need for clearer policy around the governance of general practice data and the development of clinical decision support tools. In particular, concerns have been raised about the variable quality of existing clinical decision support tools and lack of implementation considerations, which may create general distrust in electronic clinical decision support by GPs [19].

A commonly reported barrier to the use of clinical decision support systems is that of “too much information,” either in the content of a prompt or in the total volume of prompts [20,21]. This notification fatigue is an important consideration for any clinical decision support tool that activates automatically. There is a delicate balance between “active delivery” to facilitate easy interaction with a tool, and unobtrusiveness to the extent that clinicians are not aware of a tool, which is what occurred with the FHT dashboard. Clinicians in our study praised the physical size and placement of the PoC pop-up on the screen, as well as the simplicity of wording of the recommendations. Only 1 GP brought up concerns about notification fatigue, although did not report it as a barrier for their own use of the PoC tool. As more FHT modules are developed in the future, it will be vital to account for potential alert fatigue in how the pop-ups are deployed and displayed.

Lack of time has similarly been cited as a widespread barrier to clinical care for both GPs and nurses [9,18]; however, clinicians in our study did not describe time as a barrier to the use of the PoC tool. This is likely due to the speed at which they interacted with the tool, which itself was due to the usability of the tool and knowledge of the GPs. The marginal time required to interact with the tool, however, will inevitably become more significant as more FHT modules are added. It will be important for the sustainability of the FHT program to take this into account, to reduce the risk that lack of time becomes a barrier to the use of the entire PoC tool.

A study in 2015 in the United States reported barriers to the implementation of a clinical decision support tool specific to CKD, which included limited knowledge of CKD guidelines, lack of interest or confidence in technology, concerns about patient engagement, and time and competing demands [22]. In our study, clinicians’ knowledge of CKD and interest in technology for quality improvement were strong facilitators for FHT, and they were confident in discussing recommendations with their patients. Lack of knowledge in the identification and management of CKD has been cited as a general barrier to care [9]. Overall, it appears that our group of clinicians were particularly knowledgeable and proactive, and this greatly facilitated their engagement with FHT. The PoC tool had been designed with usability and clarity in mind, but these health professional characteristics were vital for uptake.

## Supplementary material

10.2196/55667 Multimedia Appendix 1 Screenshots of Future Health Today software.

10.2196/55667 Multimedia Appendix 2 Interview guide.

## Abbreviations

ACEi: angiotensin-converting enzyme inhibitor
ARB: angiotensin-receptor blocker
CKD: chronic kidney disease
CP-FIT: Clinical Performance Feedback Intervention Theory
FHT: Future Health Today
GP: general practitioner
PN: practice nurse
PoC: point of care
VicREN: Victorian Primary Care Practice-Based Research and Education Network
