# Supplementary material for: Clinical Acceptability of a Quality Improvement Program for Reducing Cardiovascular Disease Risk in People With Chronic Kidney Disease in Australian General Practice: Qualitative Study
Source: JMIR Hum Factors. 2024 Nov 13;11:e55667. doi: 10.2196/55667 (PMC11577681; doi:10.2196/55667)
Supplement: Multimedia Appendix 1 [file humanfactors-v11-e55667-s001.docx]

**Note: no screenshots include real patient names or data**

**Point of care tool**

The FHT point of care tool appears on the right side of the clinical practice software when a patient file is open. If there are active recommendations, the sidebar is orange. Clicking on the sidebar will open the recommendation.


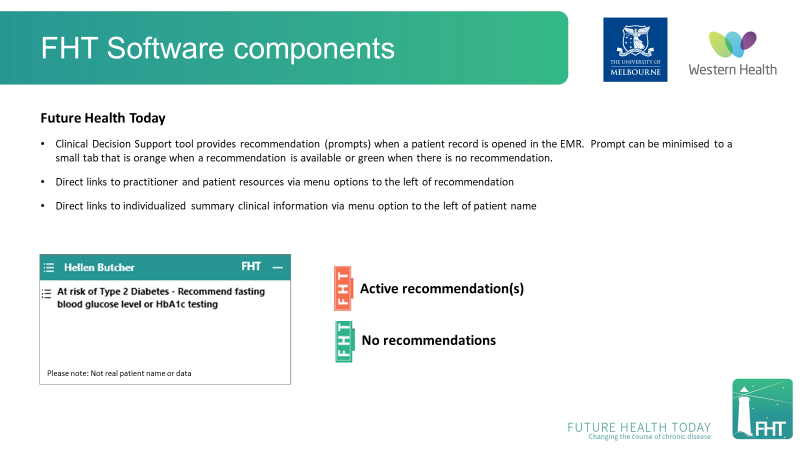


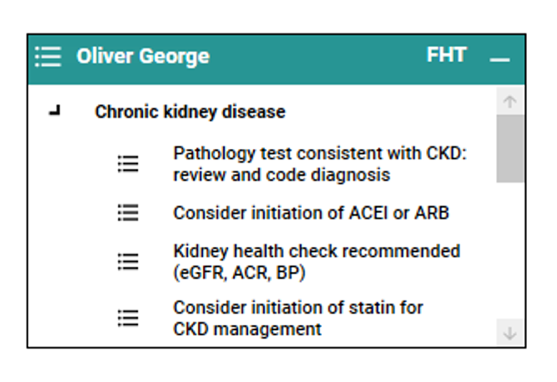

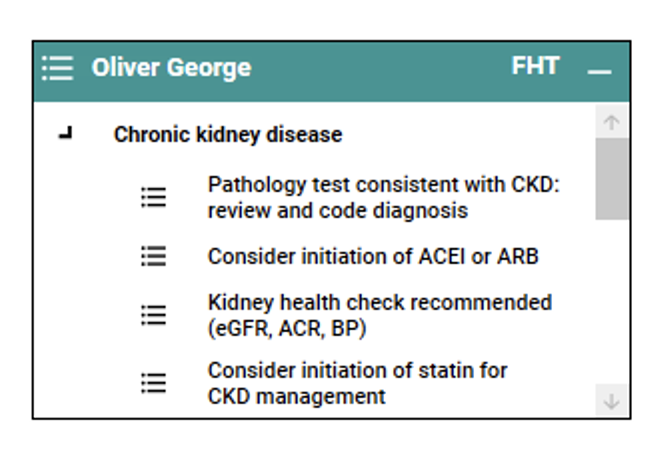

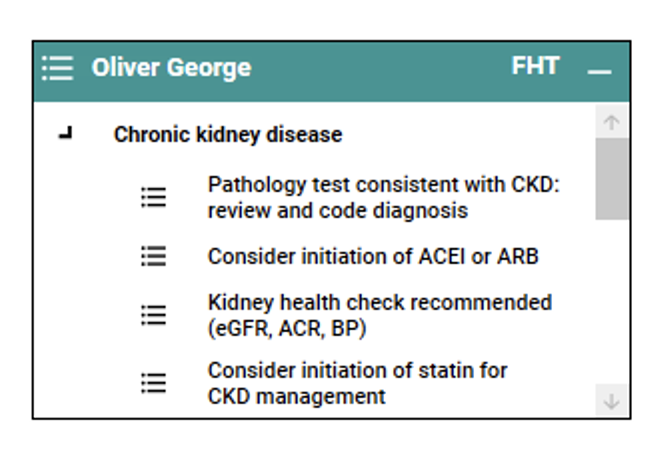


Hovering over recommendation (highlighted in grey below) reveals the clinical factors leading to the recommendation.


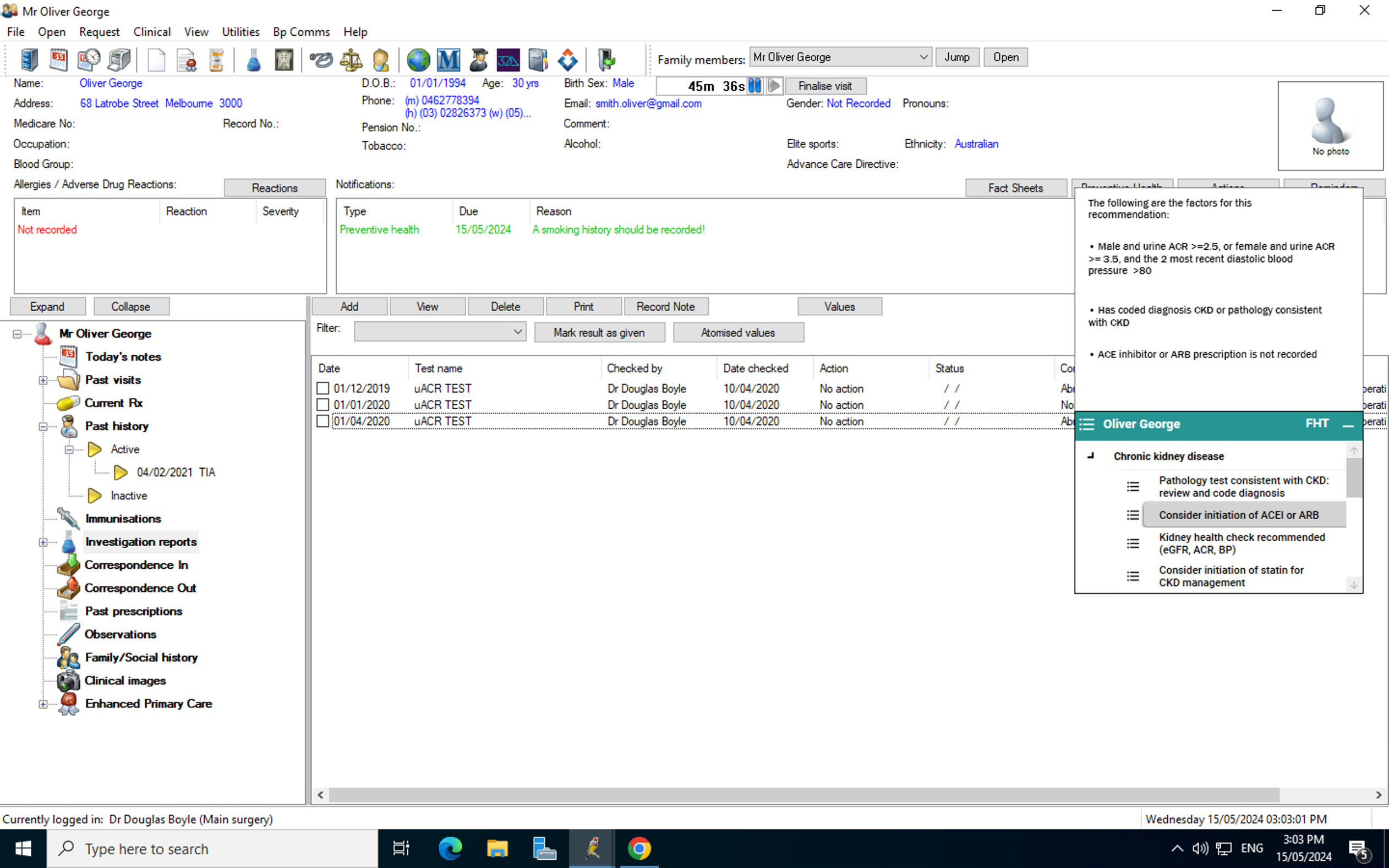

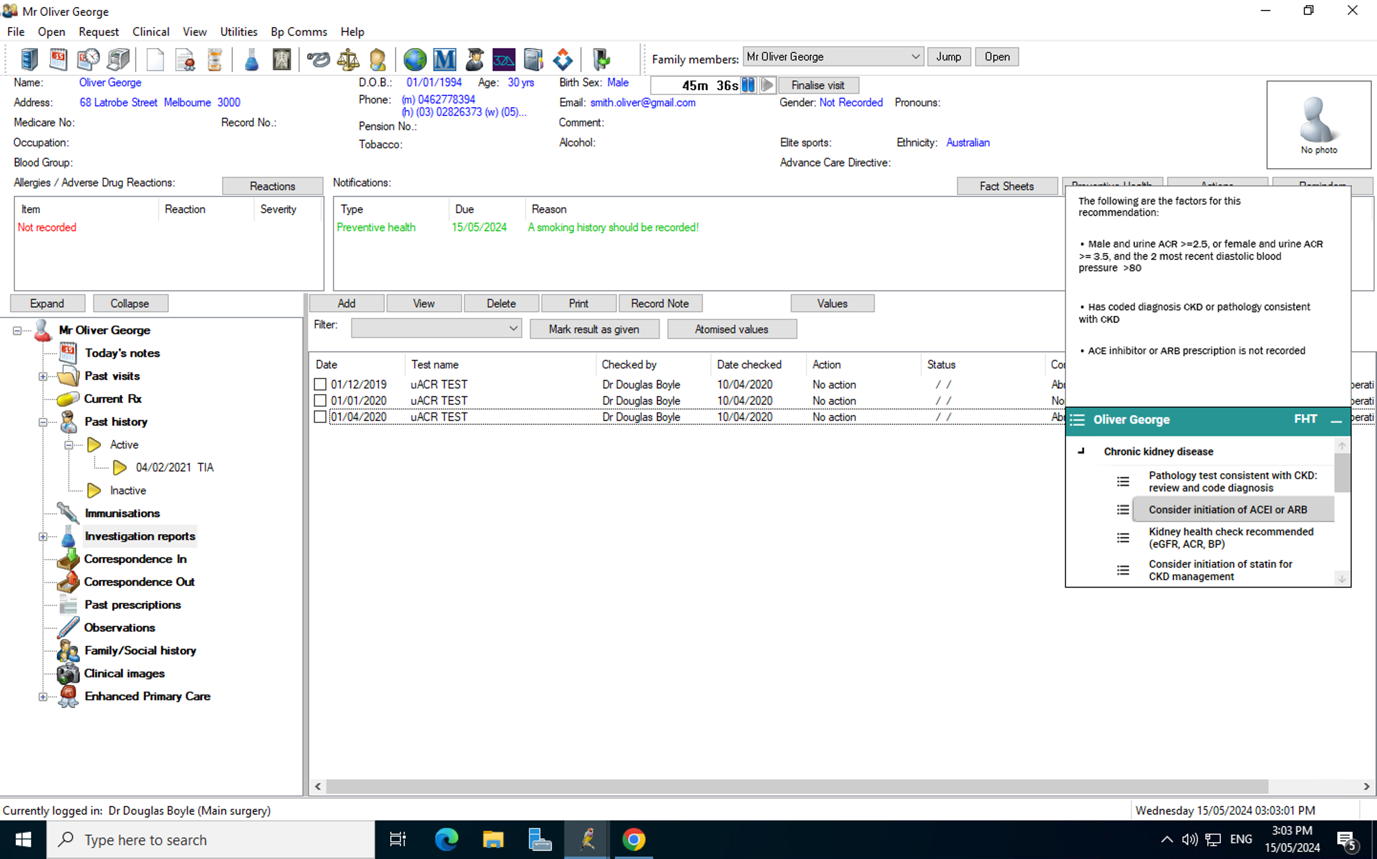

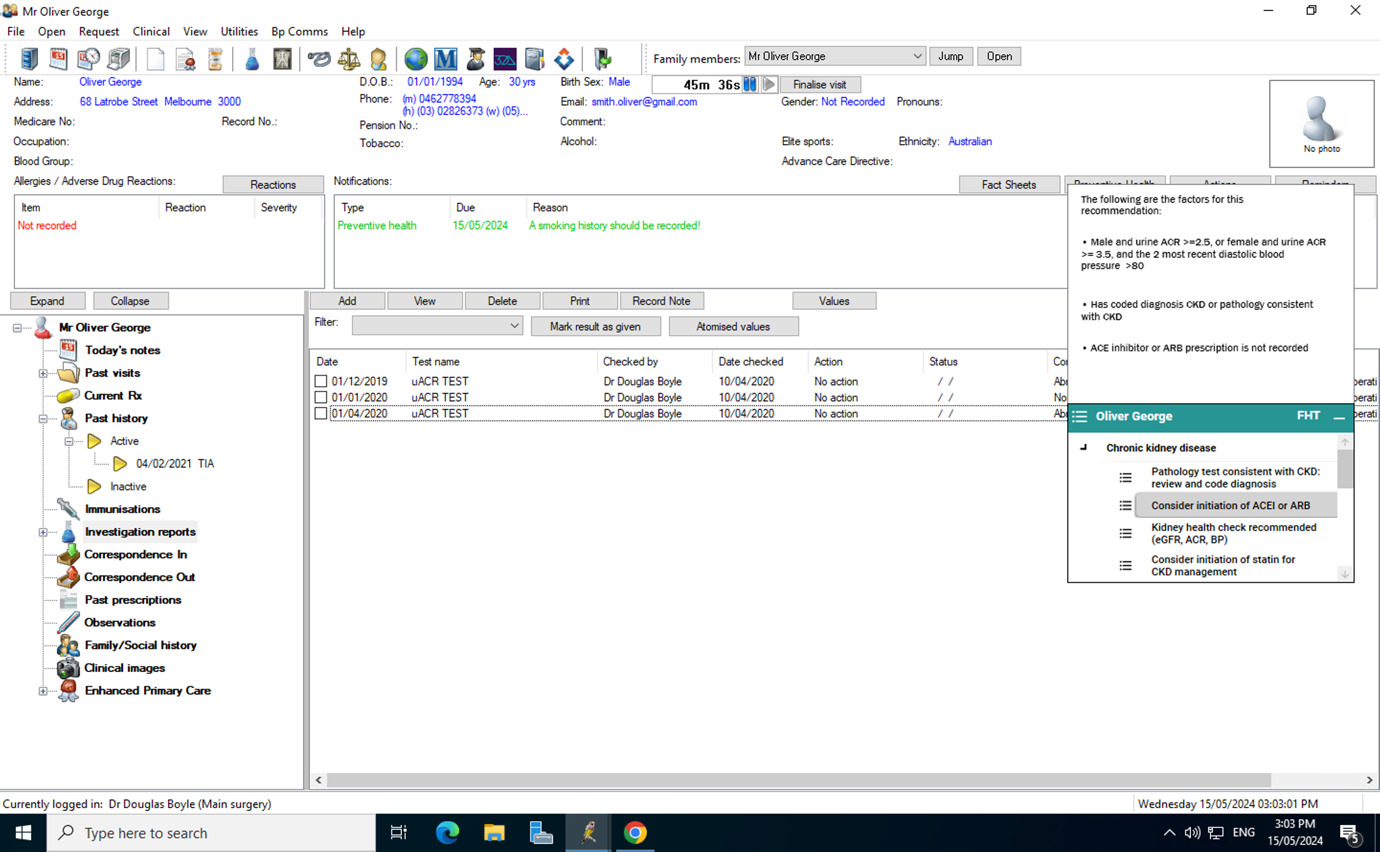

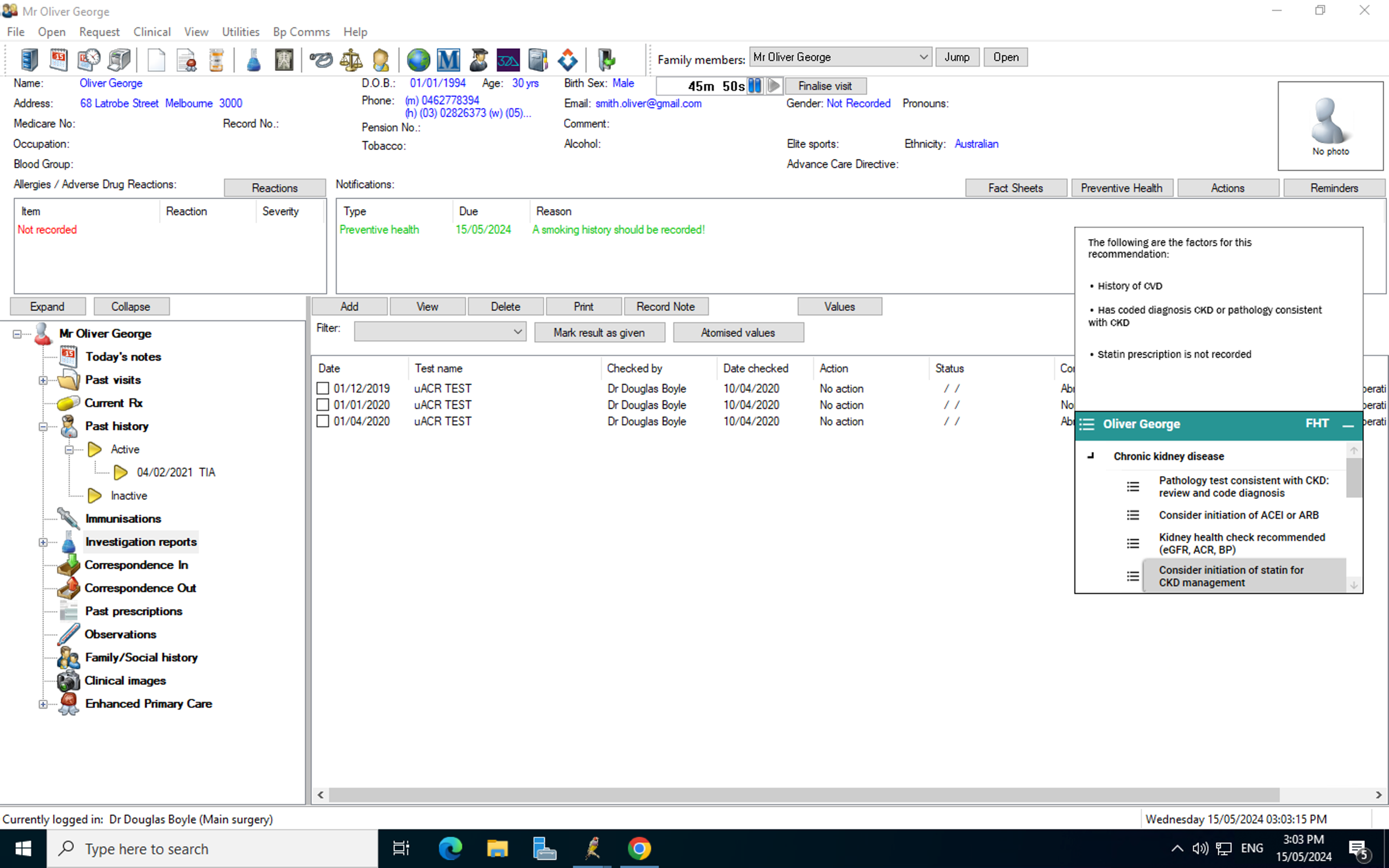

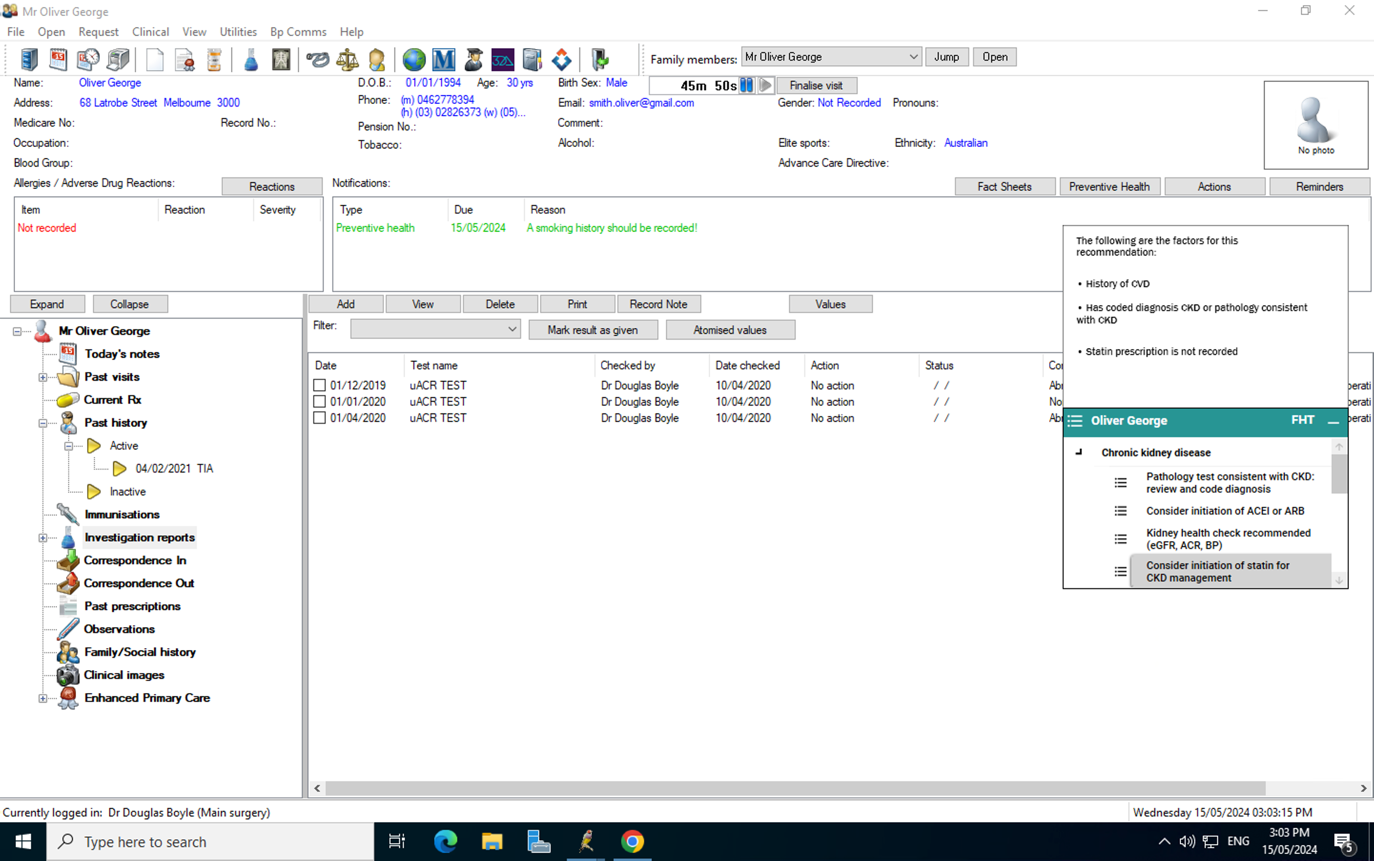

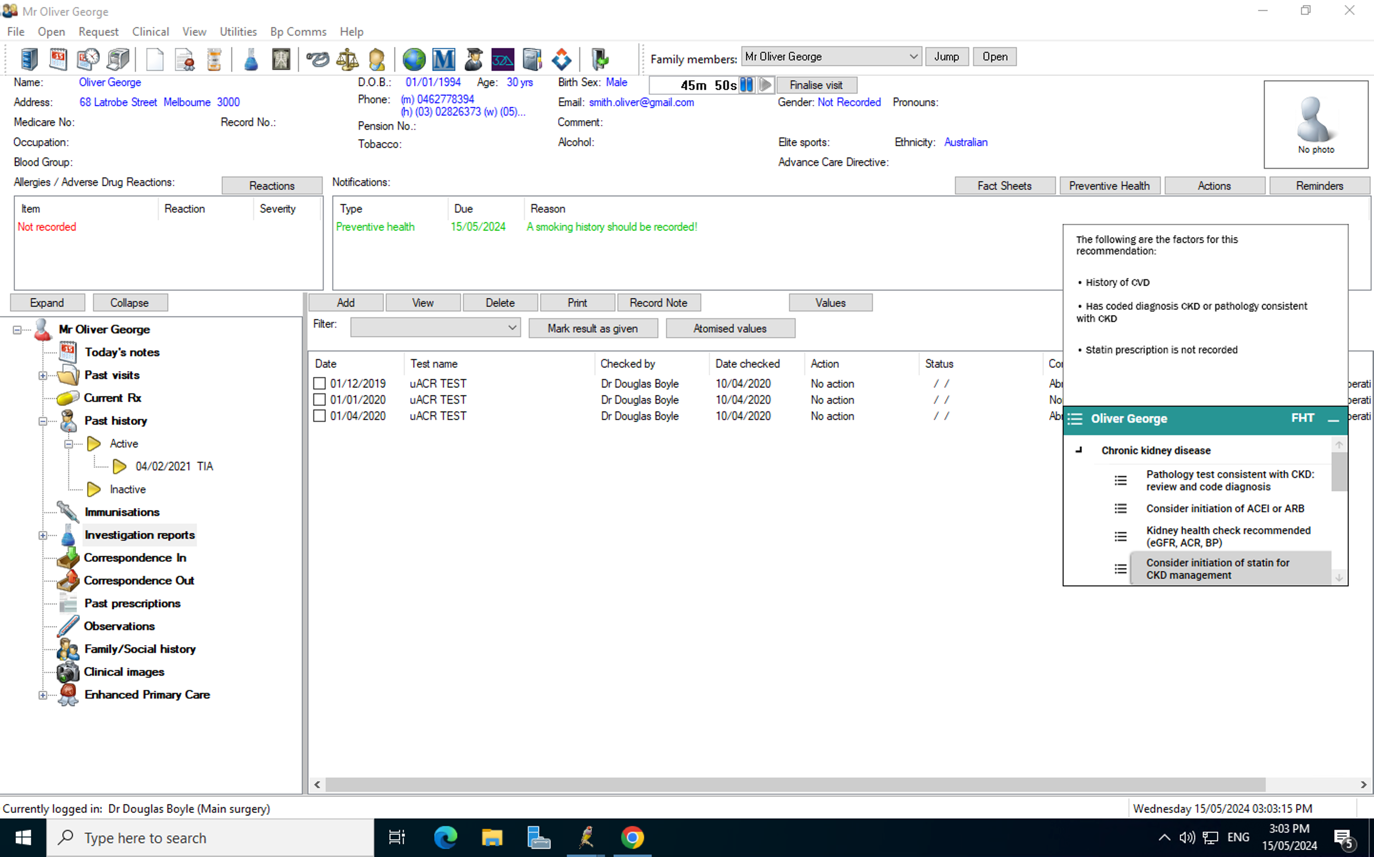


Hovering over the patient icon reveals access to resources and other actions.


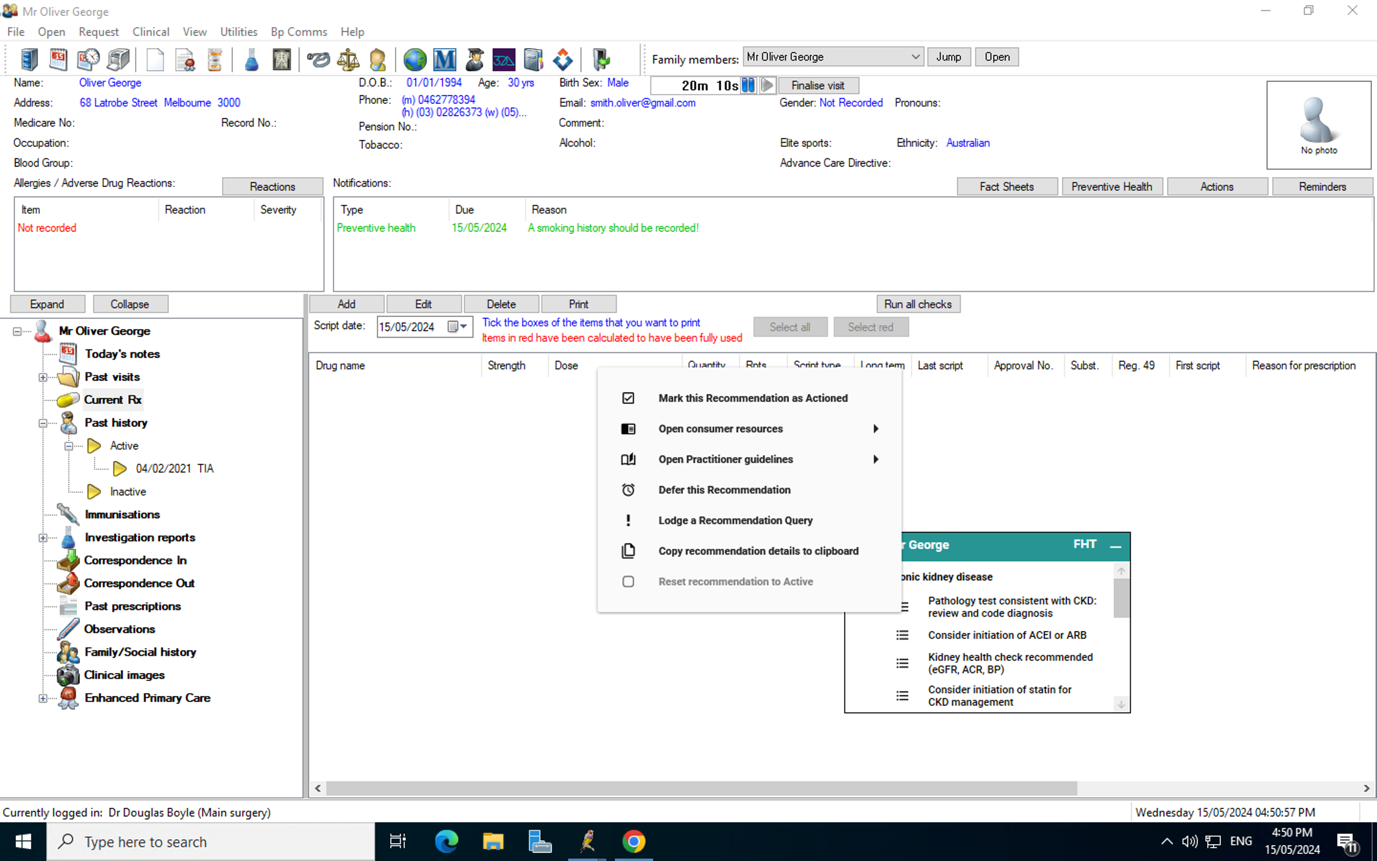

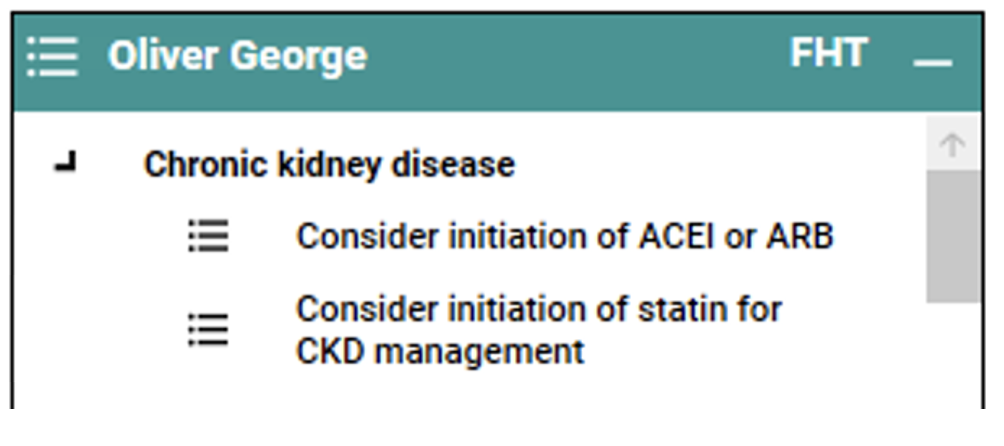


Hovering over the patient icon reveals more options, including a link the FHT home page. Clicking this opens the dashboard home page in a browser window.


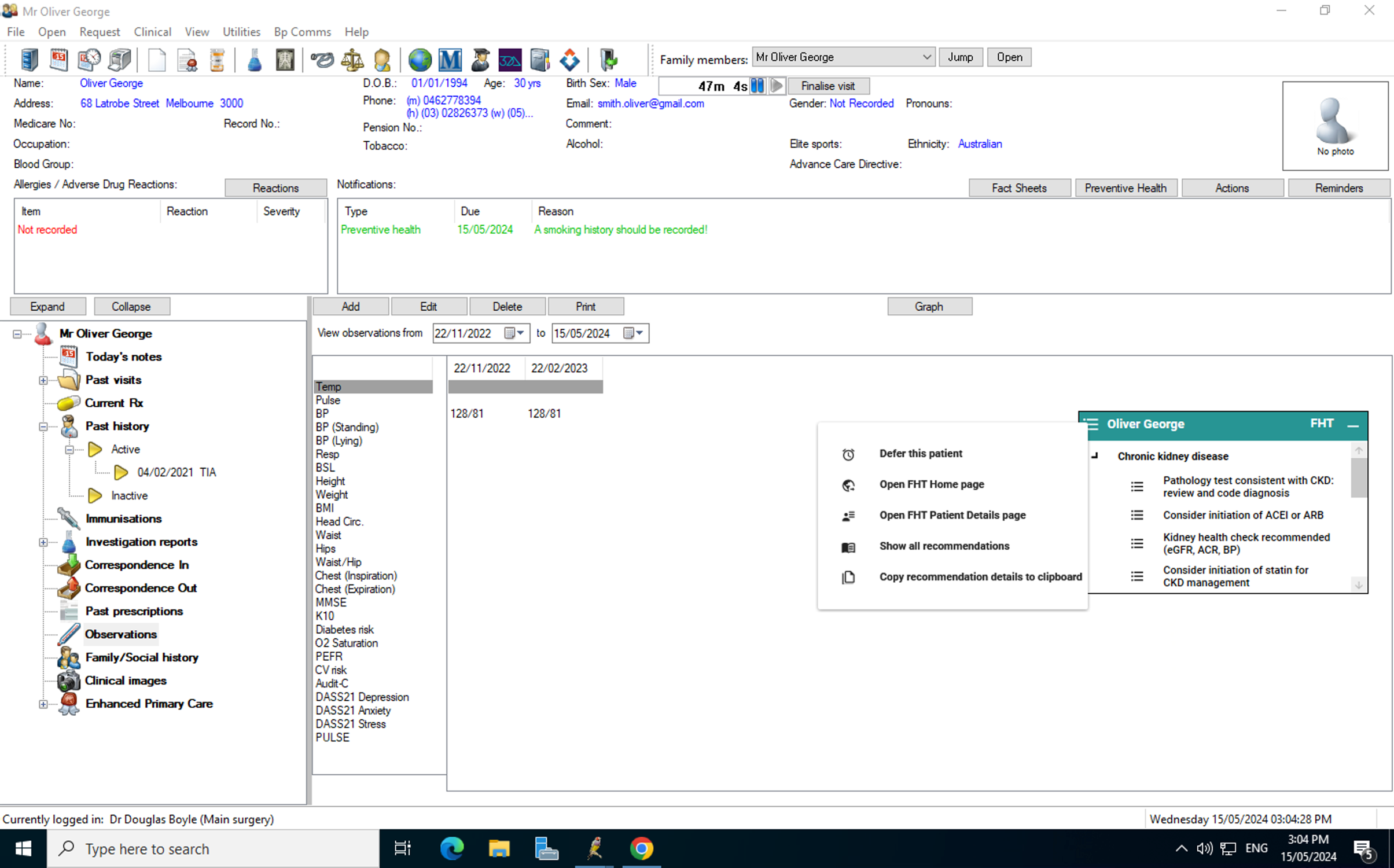

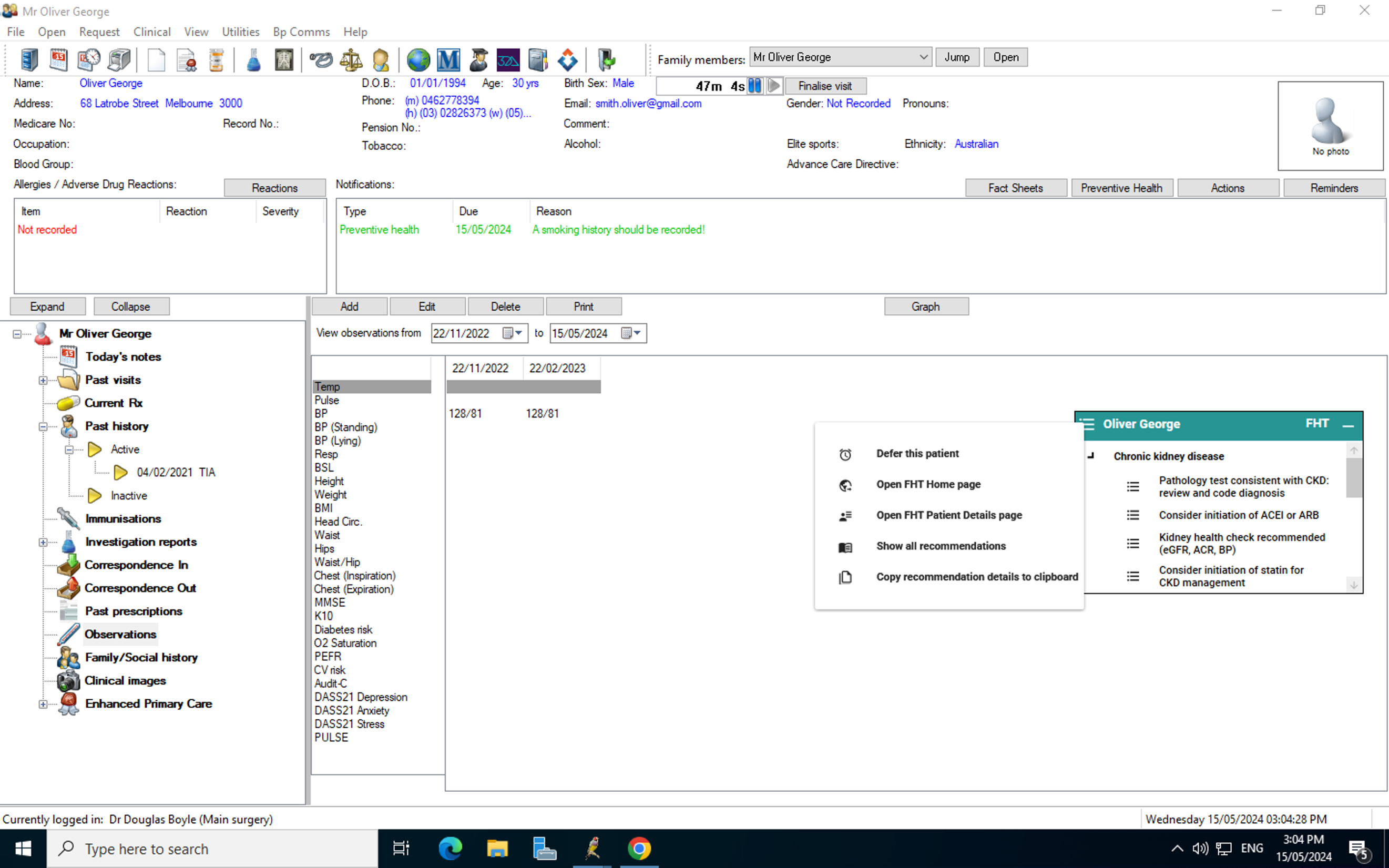

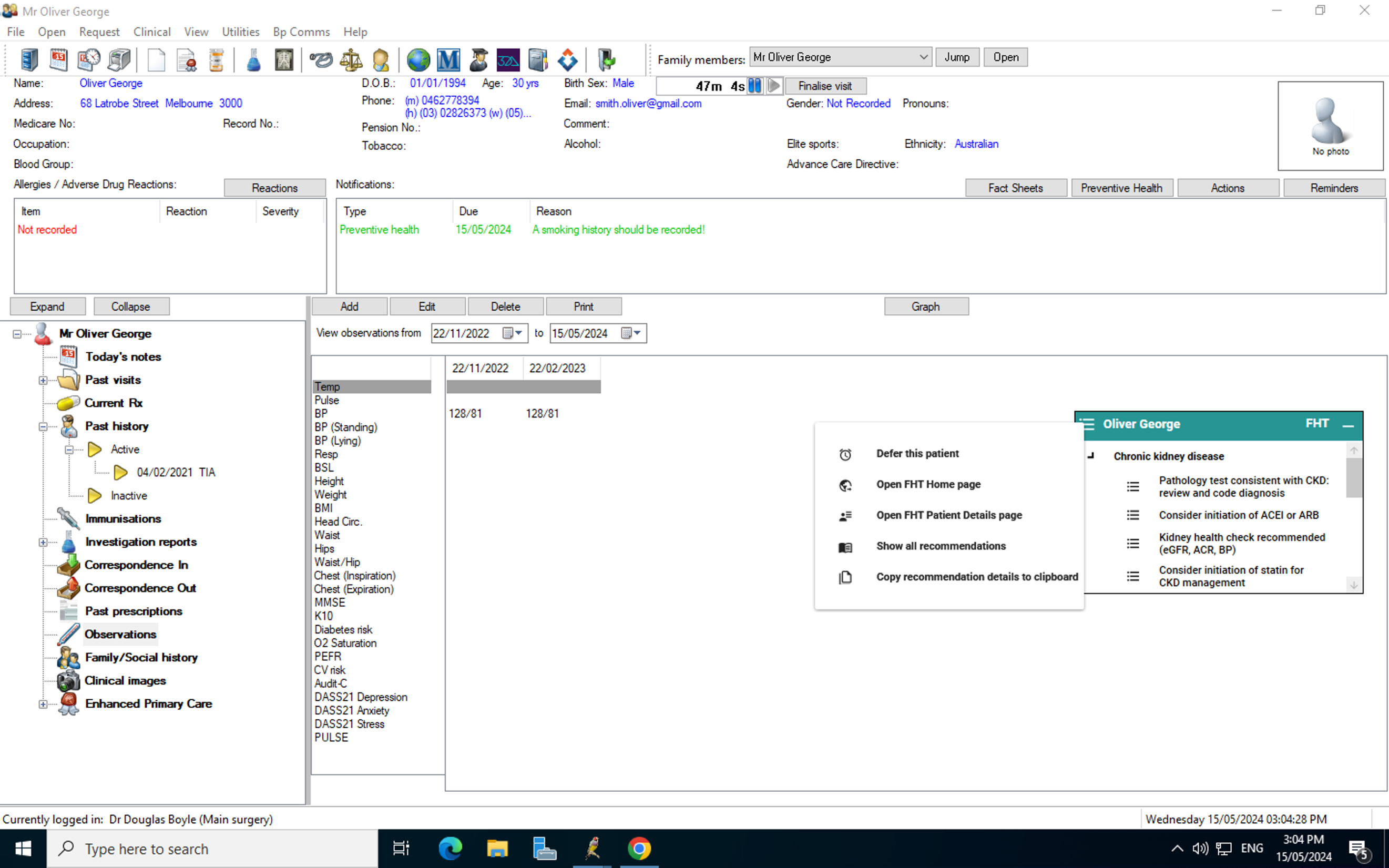

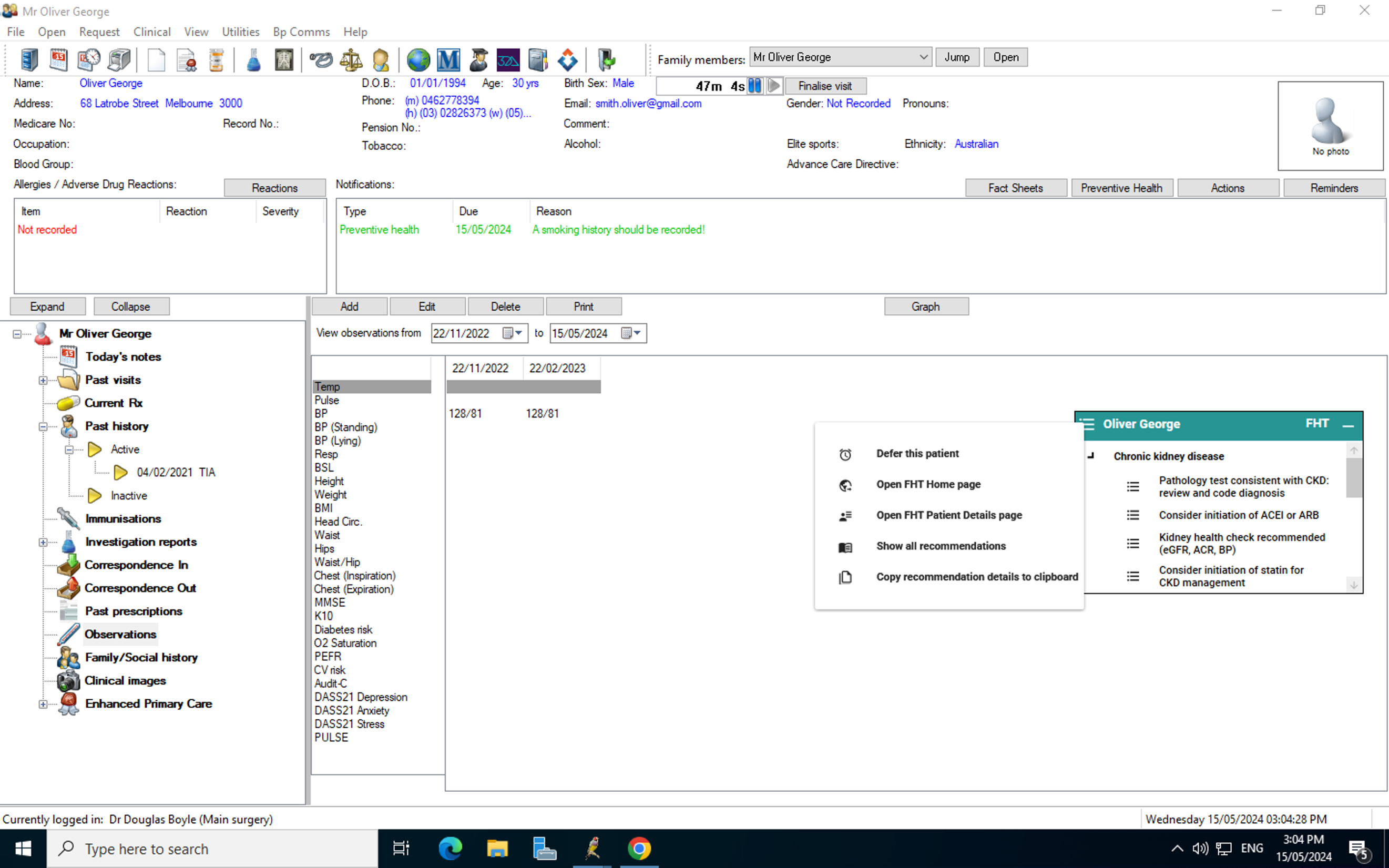


**FHT dashboard tool**

From the FHT dashboard home page, the ‘create a cohort’ function can be accessed.


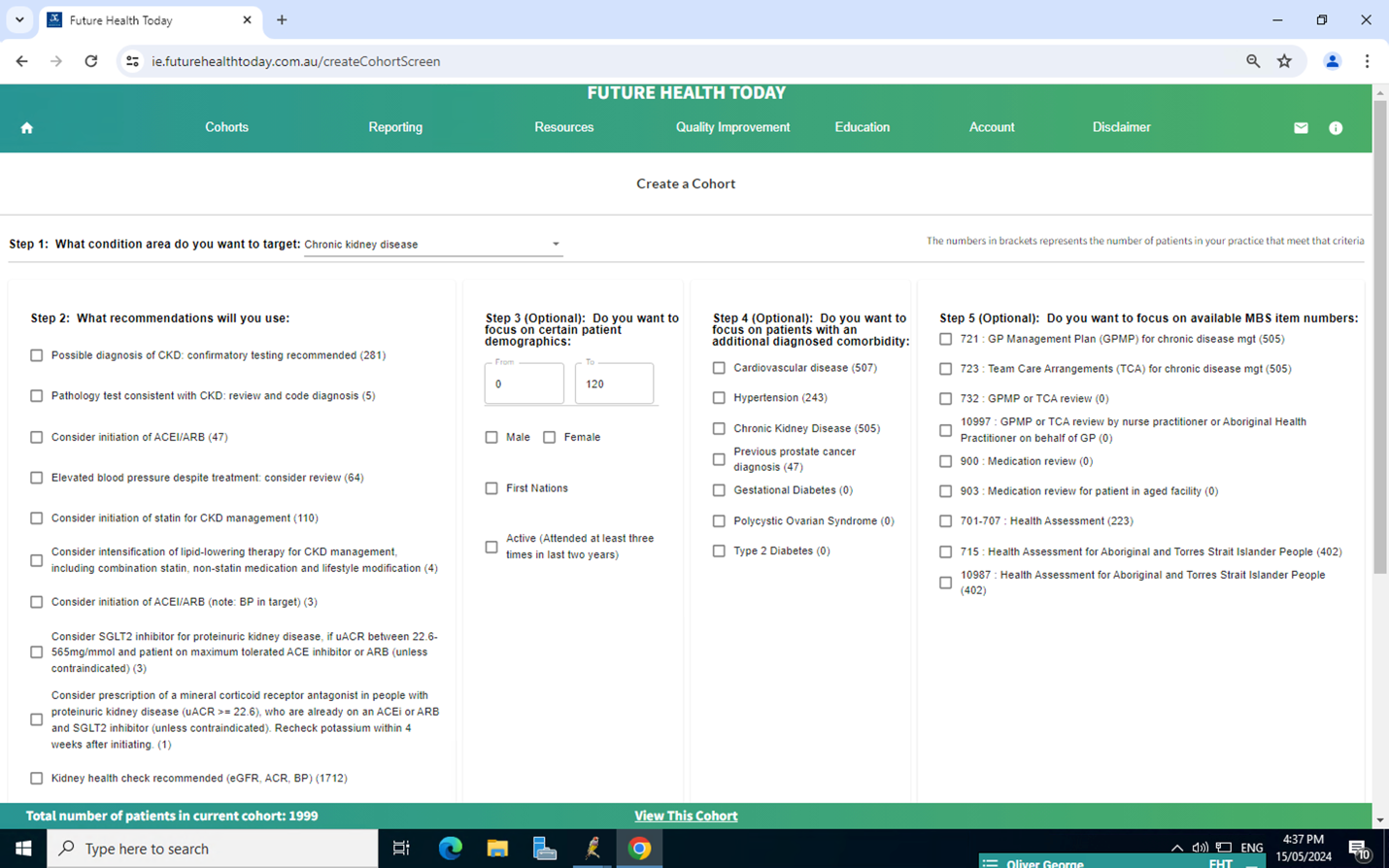


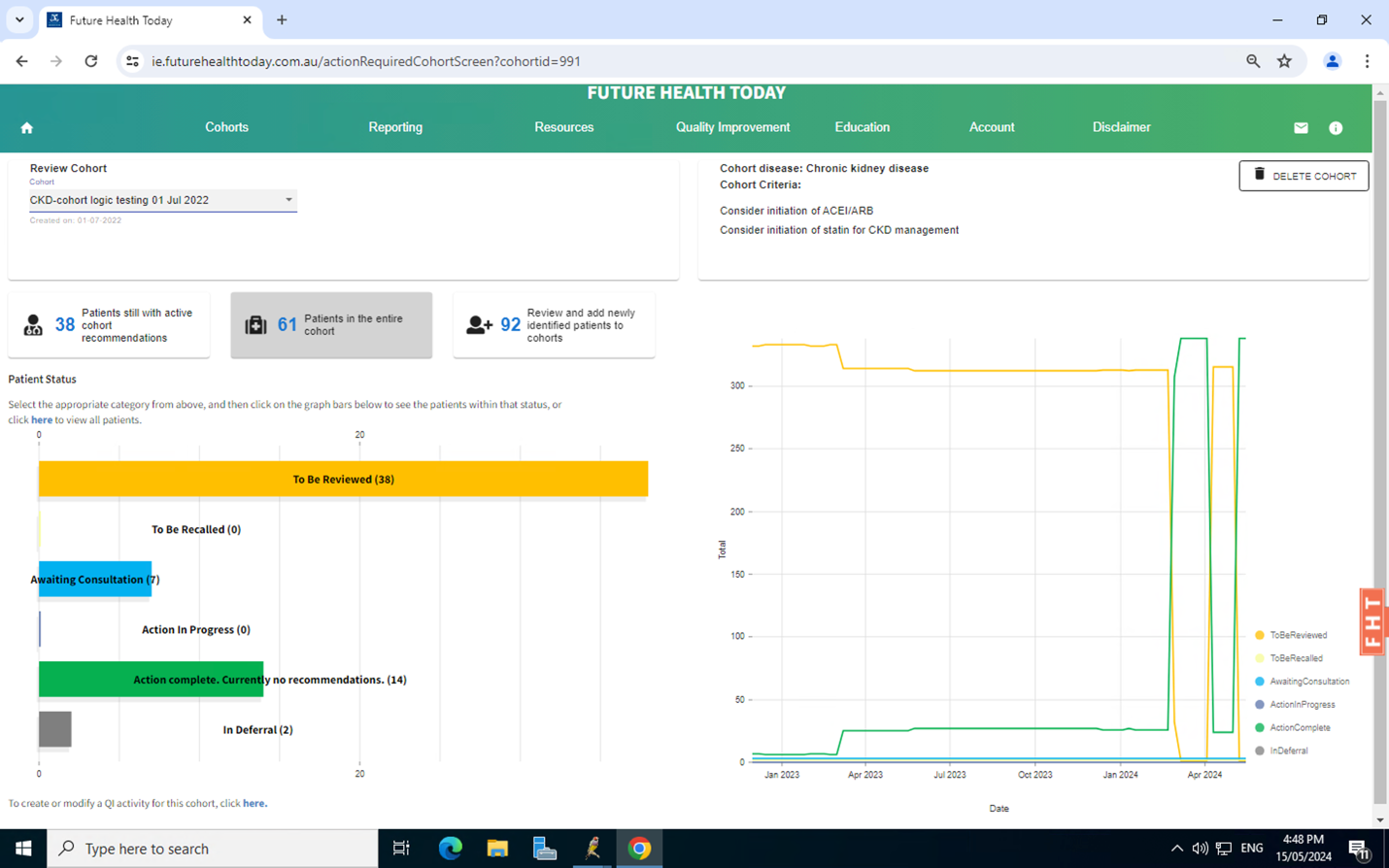
Saved cohorts can be reviewed with the ‘review cohort’ function.
