# Supplementary material for: Clinical Acceptability of a Quality Improvement Program for Reducing Cardiovascular Disease Risk in People With Chronic Kidney Disease in Australian General Practice: Qualitative Study
Source: JMIR Hum Factors. 2024 Nov 13;11:e55667. doi: 10.2196/55667 (PMC11577681; doi:10.2196/55667)
Supplement: Multimedia Appendix 2 [file humanfactors-v11-e55667-s002.docx]

## Interview schedule CKD: month 7 (interview with GPs):

**Introductions**

**Opening question:**

1. I’m talking to you because you’ve been nominated as a user of FHT. Could you quickly describe how you have been using FHT?
   - *When do you use it?*
   - *How do you use it (e.g., in the PoC, do you click on anything?)*
   - *How long does it take to read and respond to the recommendations?*
   - *Have you accessed the portal? If yes, probe when/what for?*
2. Do you think your use of FHT has changed since the start of the trial in October last year?
   - *If yes,*
     - *In what ways has your use changed?*
   - *What do you think has influenced this change?*
3. Were you aware of the short break of availability of FHT over Dec, Jan and Feb?
   - *Did the short break of availability of FHT over Dec, Jan and Feb influence your use of FHT and how?*
   - *Did this break influence your opinion of FHT and in what way?*

1. The clinical focus of FHT in your practice is CKD, how do you feel about that?
   - *Do you think it is an important area to explore?*
   - *Is it an area you feel know well?*
2. How useful do you find the recommendations?

- *In the context of your everyday practice, how useful do you think the recommendations are for identifying people who require additional management for CKD?*
- *Is FHT flagging patients that you wouldn’t have otherwise followed-up?*
- *Can you describe an experience where you found the recommendations useful?*
- *Have the recommendations influenced/changed your understanding and management of patients with CKD? (if yes, how? In what ways?)*

1. How have you addressed and discussed the FHT recommendations with your patients?
   - *Do you feel comfortable having this conversation?*
   - *Do you feel comfortable bringing this up with the patient if it is not what they came in for?*
   - *How do you frame the discussion/how have you had the conversation?*
   - *How much do you explain to patients about why they are being prescribed alternative medications?*
   - *When do you bring it up/when is it managed?*
   - *Have you shown the patient resources/visual and/ or clinical information from FHT to facilitate the discussion?*
   - *Do you need anything else to support the conversation?*
   - *Is the process any different when FHT prompts you?*
2. When are you using the point-of-care pop-up?
   - *For example, would you check it before seeing the patient?*
   - *Do you deal with the recommendation at the beginning of the consultation or try and deal with it at the end (after the presenting complaint)?*
   - *How does it interfere/affect the flow of the consultation?*
   - *Do you use it differently (e.g. more/less) on a telehealth consultation than in a face to face consultation? (and in what way is your use different)?*
   - *How often do you act on a recommendation at the point-of-care [e.g. for CKD, to prescribe]?*
   - *How often would you defer a patient? What is your process in determining if the prompt is relevant for that patient?*
   - *If they have deferred a patient or recommendation, probe for reasons why and whether it would be helpful to have a drop-down list of reasons for deferral*
   - *How often do you not action a recommendation (i.e., close the recommendation and not look at it)?*
   - *What are the main reasons that you action/don’t action a recommendation?*
3. When are you using the cohort tool (**only ask if it was indicated earlier that they are using the cohort tool**)?
   - *How often would you use the cohort tool?*
   - *Have you used the cohort tool to recall patients?*
   - *If yes, can you tell me about that process?*
   - *What is working well/what isn’t?*
   - *Does this process differ from your usual recall process?*
   - *Have the patients been receptive to being recalled? If yes/no, probe about experiences.*
4. Have you made changes to the way you work because of the prompt?
   - *If so, what changes?*
   - *Do you feel this is a useful time for the pop-up to appear and for you to interact with the prompt?*
   - *Are there any changes you would like to make to get better use out of FHT?*
5. How accurate do you think FHT is in identifying the right information?
   - *Is it flagging the patients you think it should flag?*
   - *If not, why/who/how often?*
   - *What do you do if you don’t think that the information is correct?*
   - *Do you feel able/confident in questioning the recommendation?*
   - *Are you able to verify the information provided by FHT?*
   - *Have you accessed the resources and/or supporting evidence? If yes, were they useful?*
6. Did you find any of the recommendations surprising?
   - *Have you queried or felt like you needed more information to understand any of the recommendations?*
   - *Are the recommendations consistent with your usual practice?*
   - *How confident are you in your ability to use and understand the FHT recommendations? Is there anything that would help with this process?*
7. Have you attended any of the ECHO sessions?
   - *If yes:*
   - *Did this influence how you used FHT?*
   - *Did you find the ECHO sessions a useful tool to support the use of FHT?*
   - If no:
   - *Probe why not?*
   - *Did they feel they had the knowledge/skills to use the tool and the recommendations without additional education?*
8. Has there been anything that has affected the way that you have used the tool/your ability to use the tool?
   1. Ability to get to training workshops
   2. Technical issues (including slowing of machines)
   3. Staffing issues/changeover of staff
   4. COVID and other vaccinations
   5. Other barriers that stop them from using it more

*I have one last thing to ask – would you be willing to participate in an interview with our wonderful Megan about medicolegal risk? The interview should take 15-20 minutes, like this one. ($50 voucher for participation)*

*Thanks so much for speaking with me today. We very much appreciate your time and feedback.*

## Interview schedule CKD: month 7 (interview with PNs):

**Introductions**

**Opening question:**

1. I’m talking to you because you’ve been nominated as a user of FHT. Could you quickly describe how you have been using FHT?
   - *When do you use it?*
   - *How do you use it (e.g., in the PoC, do you click on anything?)*
   - *How long does it take to read and respond to the recommendations?*
   - *Have you accessed the portal? If yes, probe what for?*
2. Do you think your use of FHT has changed since the start of the trial in October last year?
   - *If yes,*
     - *In what ways has your use changed?*
   - *What do you think has influenced this change?*
3. Were you aware of the short break of availability of FHT over Dec, Jan and Feb?
   - *Did the short break of availability of FHT over Dec, Jan and Feb influence your use of FHT and how?*
   - *Did this break influence your opinion of FHT and in what way?*
4. The clinical focus of FHT in your practice is CKD, how do you feel about that?
   - *Do you think it is an important area to explore?*
   - *Is it an area you feel know well?*
5. Have you made changes to the way you work because of FHT?
   - *If so, what changes?*
   - *Can you describe how it fits into your usual workflow (e.g., managing FHT rec’s as part of the chronic care plan)?*
   - *Do you have a system with the GPs or other practice staff on managing the FHT prompts at the point-of care?*
6. When are you using the point-of-care pop-up?
   - *For example, would you check it before seeing the patient?*
   - *Do you deal with the recommendation at the beginning of the consultation or try and deal with it at the end (after the presenting complaint)?*
   - *How does it affect the flow of the consultation?*
   - *How often do you act on a recommendation at the point-of-care to prescribe, and in what way do you act (knowing they can’t prescribe)?*
   - *How often would you defer a patient? What is your process in determining if the prompt is relevant for that patient?*
   - *If they have deferred a patient or recommendation, probe for reasons why and whether it would be helpful to have a drop-down list of reasons for deferral*
   - *How often do you not action a recommendation (i.e., close the recommendation and not look at it)?*
   - *What are the main reasons that you action/don’t action a recommendation?*
7. When are you using the cohort tool (**only ask if it was indicated earlier that they are using the cohort tool**)?
   - *How often would you use the cohort tool?*
   - *Have you used the cohort tool to recall patients?*
   - *If yes, can you tell me about that process?*
   - *What is working well/what isn’t?*
   - *Does this process differ from your usual recall process?*
   - *Have the patients been receptive to being recalled? If yes/no, probe about experiences.*
8. How have you addressed and discussed the FHT recommendations with your patients?
   - *Do you feel comfortable having this conversation?*
   - *Do you feel comfortable bringing this up with the patient if it is not what they came in for?*
   - *How do you frame the discussion/how have you had the conversation?*
   - *When do you bring it up/when is it managed?*
   - *Have you shown the patient resources/visual and/ or clinical information from FHT to facilitate the discussion?*
   - *Do you need anything else to support the conversation?*
   - *Is the process any different when FHT prompts you?*
9. How useful do you find the recommendations?

- *In the context of your everyday practice, how useful do you think the recommendations are for identifying people requiring additional medication management?*
- *Is FHT flagging patients that you wouldn’t have otherwise followed-up?*
- *Have you had an experience where you found the recommendations useful?*
- *Have the recommendations influenced/changed your understanding and management of patients with CKD? (if yes, how? In what ways?)*

1. How accurate do you think FHT is in identifying the right information?
   - *Is it flagging the patients you think it should flag?*
   - *If not, why/who/how often?*
   - *What do you do if you don’t think that the information is correct?*
   - *Do you feel able/confident in questioning the recommendation?*
   - *Are you able to verify the information provided by FHT?*
   - *Have you accessed the resources and/or supporting evidence? If yes, were they useful?*
2. Did you find any of the recommendations surprising?
   - *Have you queried or felt like you needed more information to understand any of the recommendations?*
   - *Are the recommendations consistent with your usual practice?*
   - *How confident are you in your ability to use and understand the FHT recommendations? Is there anything that would help with this process?*
3. Have you attended any of the ECHO sessions?
   - *If yes:*
   - *Did this influence how you used FHT?*
   - *Did you find the ECHO sessions a useful tool to support the use of FHT?*
   - If no:
   - *Probe why not?*
   - *Did they feel they had the knowledge/skills to use the tool and the recommendations without additional education?*
4. Has there been anything that has affected the way that you have used the tool/your ability to use the tool?
   1. Ability to get to training workshops
   2. Technical issues (including slowing of machines)
   3. Staffing issues/changeover of staff
   4. COVID and other vaccinations
   5. Other barriers that stop them from using it more

*I have one last thing to ask – would you be willing to participate in an interview with our wonderful Megan about medicolegal risk? The interview should take 15-20 minutes, like this one. ($50 voucher for participation)*

*Thanks so much for speaking with me today. We very much appreciate your time and feedback.*
